# Supplementary material for: Protocol for educational programs on infection prevention/control for medical and healthcare student: A systematic review and meta-analysis
Source: PLoS One. 2022 Oct 27;17(10):e0276851. doi: 10.1371/journal.pone.0276851 (PMC9612507; doi:10.1371/journal.pone.0276851)
Supplement: Attachment — Submitted filename: Response_to_Reviewers.docx [file pone.0276851.s001.docx]

Oct 11, 2022

Sawsan Abuhammad

Academic Editor

*PLOS ONE*

Dear Sawsan Abuhammad,

Thank you for the thoughtful and constructive feedback you provided regarding our manuscript, “Systematic review protocol for educational program on infection prevention/control for students in medical and healthcare schools.” We agree with your suggestions regarding ways to improve our manuscript, and have, therefore, revised its content accordingly (in the document “Revised Manuscript with Track Changes”). Additionally, we have ensured that the manuscript’s format and contents are in accordance with the journal’s guidelines. We are certain that you will find that the most recent version of our manuscript addresses the main issues that you have indicated.

Again, thank you for giving us the opportunity to strengthen our manuscript through your valuable comments and queries. We have worked hard to incorporate your feedback and hope that these revisions persuade you to accept our submission.

With these changes to our manuscript, we, hereby, resubmit it for evaluation. Thank you, once again, for considering our paper.

Sincerely,

Naonori Tashiro

Department of Physical Therapy, Showa University School of Nursing and Rehabilitation Sciences

1865 Tokaichibacho, Midori-ku, Yokohama-shi, Kanagawa 226-8555, Japan

Tel: 81-45-985-6500, Fax: +81-45- 985-6557

Email: [tashiro@cmed.showa-u.ac.jp](mailto:tashiro@cmed.showa-u.ac.jp)

Response to Journal Requirements:

Thank you very much for providing important comments. We are thankful for the time and energy you expended. Our responses to the referees’ comments are as follow:

1. Please ensure that your manuscript meets PLOS ONE's style requirements, including those for file naming. The PLOS ONE style templates can be found at

<https://journals.plos.org/plosone/s/file?id=wjVg/PLOSOne_formatting_sample_main_body.pdf> and

<https://journals.plos.org/plosone/s/file?id=ba62/PLOSOne_formatting_sample_title_authors_affiliations.pdf>

**RESPONSE: Thank you for pointing this out. We have formatted the manuscript in accordance with the journal guidelines.**

2. Thank you for stating in your Funding Statement: "This study was supported by School of Nursing and Rehabilitation Sciences Showa University Research Fund (Grant Numbers 2022No.3: AY)."

Please provide an amended statement that declares *all* the funding or sources of support (whether external or internal to your organization) received during this study, as detailed online in our guide for authors at http://journals.plos.org/plosone/s/submit-now. Please also include the statement “There was no additional external funding received for this study.” in your updated Funding Statement. Please include your amended Funding Statement within your cover letter. We will change the online submission form on your behalf.

**RESPONSE: Thank you for pointing this out. This study received no additional external funding, therefore, we have added a sentence regarding this (*line 284).***

3. Thank you for stating in your Funding Statement: "This study was supported by School of Nursing and Rehabilitation Sciences Showa University Research Fund (Grant Numbers 2022No.3: AY)."

Please state what role the funders took in the study. If the funders had no role, please state: ""The funders had no role in study design, data collection and analysis, decision to publish, or preparation of the manuscript."" If this statement is not correct you must amend it as needed. Please include this amended Role of Funder statement in your cover letter; we will change the online submission form on your behalf.

**RESPONSE: Thank you for pointing this out.** **The funders had no role in the study design, data collection and analysis, decision to publish, or preparation of the manuscript; therefore, per your comment, we have stated this in the manuscript (*lines 282–284)* and covering letter.**

4. Thank you for stating the following in your Competing Interests section: "The authors declare no competing interests."

Please complete your Competing Interests on the online submission form to state any Competing Interests. If you have no competing interests, please state ""The authors have declared that no competing interests exist."", as detailed online in our guide for authors at <http://journals>.plos.org/plosone/s/submit-now This information should be included in your cover letter; we will change the online submission form on your behalf.

**RESPONSE: Thank you for pointing this out. Per your comment, we have included a sentence declaring that no competing interests exist in the cover letter document labeled “Revised cover letter.”**

5. Please review your reference list to ensure that it is complete and correct. If you have cited papers that have been retracted, please include the rationale for doing so in the manuscript text, or remove these references and replace them with relevant current references. Any changes to the reference list should be mentioned in the rebuttal letter that accompanies your revised manuscript. If you need to cite a retracted article, indicate the article’s retracted status in the References list and also include a citation and full reference for the retraction notice.

**RESPONSE: Thank you for pointing this out.** **We have re-checked the reference list and the text.**

Response to Reviewer #1

We are grateful for this kind comment. Our responses to the referees’ comments are as follow:

1. Does the manuscript provide a valid rationale for the proposed study, with clearly identified and justified research questions?

The research question outlined is expected to address a valid academic problem or topic and contribute to the base of knowledge in the field.

Reviewer #1: Partly

2. Is the protocol technically sound and planned in a manner that will lead to a meaningful outcome and allow testing the stated hypotheses?

The manuscript should describe the methods in sufficient detail to prevent undisclosed flexibility in the experimental procedure or analysis pipeline, including sufficient outcome-neutral conditions (e.g. necessary controls, absence of floor or ceiling effects) to test the proposed hypotheses and a statistical power analysis where applicable. As there may be aspects of the methodology and analysis which can only be refined once the work is undertaken, authors should outline potential assumptions and explicitly describe what aspects of the proposed analyses, if any, are exploratory.

Reviewer #1: Partly

3. Is the methodology feasible and described in sufficient detail to allow the work to be replicable?

Reviewer #1: No

**RESPONSE: Thank you for your comment. We have discussed the feasibility of the methodology and described it in sufficient detail in our response to “6. Review Comments to the Author.”**

4. Have the authors described where all data underlying the findings will be made available when the study is complete?

The PLOS Data policy requires authors to make all data underlying the findings described in their manuscript fully available without restriction, with rare exception, at the time of publication. The data should be provided as part of the manuscript or its supporting information, or deposited to a public repository. For example, in addition to summary statistics, the data points behind means, medians and variance measures should be available. If there are restrictions on publicly sharing data—e.g. participant privacy or use of data from a third party—those must be specified.

Reviewer #1: Yes

5. Is the manuscript presented in an intelligible fashion and written in standard English?

PLOS ONE does not copyedit accepted manuscripts, so the language in submitted articles must be clear, correct, and unambiguous. Any typographical or grammatical errors should be corrected at revision, so please note any specific errors here.

Reviewer #1: Yes

6. Review Comments to the Author

Please use the space provided to explain your answers to the questions above and, if applicable, provide comments about issues authors must address before this protocol can be accepted for publication. You may also include additional comments for the author, including concerns about research or publication ethics.

You may also provide optional suggestions and comments to authors that they might find helpful in planning their study.

(Please upload your review as an attachment if it exceeds 20,000 characters)

Reviewer #1:

- The research question needs to be clearly defined.

**RESPONSE:** **We agree with your comment. Therefore, we have added the following sentence (*lines 110–113).***

**Our systematic review and meta-analysis’ research question is: What enhances learning satisfaction and the efficacy of simulation-based infection prevention/control education, compared to classical education, among students who are enrolled in medical, nursing, rehabilitation, and other related courses?**

- Literature search and study selection: Just two bibliographic databases were included. Typically, three bibliographic databases should be used. In order to secure proper basis for evidence-based research, it is essential to perform a broad search that includes as many studies as possible that meet the inclusion and exclusion criteria.

**RESPONSE:** **Thank you for your suggestion.** **Accordingly, we added one more database—Scopus.** **Therefore, we extended the search period from 1990 (January) to 2022 (September).** **Details regarding this have been added to manuscript *(lines 50–51, 114–115, and 158).***

- Inclusion criteria/ Types of participants: I think the inclusion of studies has biases. It is not comprehensive. Graduate students not included. However, many studies conducted on graduate students with simulation lab. Also, why medicine students and pharmacy students not included, this could yield misleading results.

**RESPONSE:** **Thank you for your suggestion. We agree with you. Therefore, we have added the following sentence (*lines 135–138).***

**The included studies will comprise participants who are undergraduate and graduate students enrolled in medical and healthcare-related occupational courses (medicine, dentistry, nursing, physical therapy, occupational therapy, pharmacy, and other healthcare-related fields).**

- Guidelines for reporting meta-analyses of RCTs were not presented clearly.

**RESPONSE:** **We agree with your assessment.** **Accordingly, we have revised the manuscript (*lines 200–207 and 213–217*).**

7. PLOS authors have the option to publish the peer review history of their article (what does this mean?). If published, this will include your full peer review and any attached files.

If you choose “no”, your identity will remain anonymous but your review may still be made public.

Do you want your identity to be public for this peer review? For information about this choice, including consent withdrawal, please see our Privacy Policy.

Reviewer #1: Yes: Ala'a Fawwaz Dalky
